# Sulprostone-Induced Gastric Dysrhythmia in the Ferret: Conventional and Advanced Analytical Approaches

**DOI:** 10.3389/fphys.2020.583082

**Published:** 2021-01-08

**Authors:** Zengbing Lu, Yu Zhou, Longlong Tu, Sze Wa Chan, Man P. Ngan, Dexuan Cui, Yuen Hang Julia Liu, Ianto Bosheng Huang, Jeng S. C. Kung, Chung Man Jessica Hui, John A. Rudd

**Affiliations:** ^1^School of Biomedical Sciences, Faculty of Medicine, The Chinese University of Hong Kong, Shatin, Hong Kong; ^2^School of Health Sciences, Caritas Institute of Higher Education, Tseung Kwan O New Town, Hong Kong; ^3^Institute of Future Cities, The Chinese University of Hong Kong, Shatin, Hong Kong; ^4^Laboratory Animal Services Centre, The Chinese University of Hong Kong, Shatin, Hong Kong

**Keywords:** sulprostone, ferret, gastric myoelectric activity, running spectrum analysis, detrended fluctuation analysis, entropy

## Abstract

Nausea and emesis resulting from disease or drug treatment may be associated with disrupted gastric myoelectric activity (GMA). Conventional analytical techniques can determine the relative degrees of brady-, normo-, and tachygastric power, but lose information relative to the basic slow wave shape. The aim of the present study was to investigate the application of advanced analytical techniques in the analysis of disrupted GMA recorded after administration of sulprostone, a prostaglandin E_3__/__1_ agonist, in ferrets. Ferrets were implanted with radiotelemetry devices to record GMA, blood pressure, heart rate (HR) and core body temperature 1 week before the administration of sulprostone (30 μg/kg) or vehicle (saline, 0.5 mL/kg). GMA was initially analyzed using fast Fourier transformations (FFTs) and a conventional power partitioning. Detrended fluctuation analysis (DFA) was also applied to the GMA recordings to reveal information relative to the fluctuation of signals around local trends. Sample entropy (SampEn) analysis was used for examining the regularity of signals. Conventional signal processing techniques revealed that sulprostone increased the dominant frequency (DF) of slow waves, with an increase in the percentage power of the tachygastric range and a decrease in the percentage power of the normogastric range. DFA revealed that sulprostone decreased the fluctuation function, indicative of a loss of the variability of GMA fluctuations around local trends. Sulprostone increased SampEn values, indicating a loss of regularity in the GMA data. Behaviorally, sulprostone induced emesis and caused defecation. It also increased blood pressure and elevated HR, with an associated decrease in HR variability (HRV). Further analysis of HRV revealed a decrease in both low-frequency (LF) and high-frequency (HF) components, with an overall increase in the LF/HF ratio. Sulprostone did not affect core body temperature. In conclusion, DFA and SampEn permit a detailed analysis of GMA, which is necessary to understand the action of sulprostone to modulate gastric function. The action to decrease HRV and increase the LF/HF ratio may be consistent with a shift toward sympathetic nervous system dominance, commonly seen during nausea.

## Introduction

Gastric dysfunction caused by altered motility occurs in a number of diseases and in response to drug treatments that cause nausea (Sanger et al., 2013). In situations of unexplained nausea and emesis, electrogastrography may be used to examine the electrical pacing of the stomach (Koch, 1989; Parkman et al., 2003). This technique records gastric myoelectric activity (GMA), which comprises low-frequency slow waves and neuronal spike activity. Slow waves originate from the interstitial cells of Cajal (ICCs), which show a specific distribution, arrangement and cell shape depending on their location within various regions and tissue layers of the gastrointestinal tract (Komuro, 2006). In healthy individuals, the frequency at which the power spectrum of slow waves with the highest power occurs approximately three times per minute; this is termed the dominant frequency (DF). During nausea, the frequency and power of these waves may increase or decrease, and other disordered waves may occur, leading to dysrhythmia. To classify the electrical state of the stomach as dysrhythmic, clinicians may observe a loss of normal rhythm either quantitatively, changes in DF, or qualitatively, by looking at signal shapes or inspecting a running spectral analysis (RSA) of data (Verhagen et al., 1999).

Using state-of-the-art radiotelemetric technology, our previous studies successfully recorded the GMA from freely moving ferrets, shrews and mice (Percie du Sert et al., 2010; Lu et al., 2014; Tu et al., 2017; Wang et al., 2018). In the ferrets, analysis of the DF and power partition of GMA revealed that GMA could be disrupted by emetics such as apomorphine (Percie du Sert et al., 2009a), cisplatin (Percie du Sert et al., 2009a), and exendin-4 (Lu et al., 2017b). In shrews, disrupted GMA was observed in animals following motion (Percie du Sert et al., 2010; Rudd et al., 2018). MFDFA has been used to analyze stationary or non-stationary time series in electromyography (EMG), electroencephalography (EEG), electrocardiography (ECG), gait variability, glycemic variability and respiration (Ihlen, 2012), in which signal shapes change in complexity. However, the correct implementation of the MFDFA assumes the power law can be found in the scaling behavior of fluctuation function and the detected multifractality should be examined by surrogate tests (Racz et al., 2019). Here, we study the series by detrended fluctuation analysis (DFA), which is a special case of MFDFA by considering only the second moment of the fluctuation function. Theoretically, the scaling power estimated by DFA can be connected to the Hurst exponent for quantifying long-range correlation. Because of its effectiveness and efficiency, DFA is recommended as the most appropriate method among many different methods to estimate the Hurst exponent, especially when the functional form of the trend is not *a priori* known (Bashan et al., 2008). Although DFA studies mainly focus on the scaling behavior via estimating the scaling power, such power actually contains part of the information about the fluctuation function (Peng et al., 1994; Kantelhardt et al., 2001). As pointed out by Kantelhardt et al. (2001), the fluctuation function is the average over the fluctuations around the local trend in each segment, which plays a role similar to variance to measure the variability of the series around the local trend. It should be emphasized that even when the fluctuation function turns out to not follow the power-law scaling behavior, the information about the variability contained in the fluctuation function at each scale is still valid. In this study, we therefore focus on the values of fluctuation function but not the DFA exponent based on its scaling behavior.

The concept of entropy has been invoked to measure irregularity in complex systems, which can usually be characterized using time series. Physiological signals such as heart rate (HR), blood pressure (BP) and respiratory variability are regulated by multiple control mechanisms, so they may constitute a complex system from the perspective of information content (Castiglioni et al., 2013; Wu et al., 2013; Diab et al., 2014; Haeufle et al., 2014). Many different definitions of entropy have been proposed, but the term is commonly defined as the rate of information production, with larger values indicating more irregularity (Costa et al., 2002). Entropy analysis has been applied to electrogastrogram (EGG) recordings in man. Peupelmann et al. used an approximate entropy (ApEn) approach to analyze EGG data from patients diagnosed with schizophrenia and found that the ApEn level of GMA during tachygastria after a test meal was higher than in controls (Peupelmann et al., 2009). A similar study showed that patients diagnosed with major depression had higher ApEn levels than controls, as well as tachygastria (Quick et al., 2010). An elevated ApEn level indicates increased complexity and dysregulation of GMA. Despite the wide application of the ApEn in physiological studies, it is actually prone to noisy or the short length of the signal (Richman and Moorman, 2000). Because of this drawback, a slightly different analytical approach, i.e., the sample entropy (SampEn), was introduced by Richman and Moorman (2000) to determine system complexity. In this study, we employed the improved entropy analysis, i.e., the SampEn analysis.

The aim of the present study was to apply conventional and advanced signal processing techniques to GMA data generated from another emetic challenge, sulprostone, that disrupts gastric function and causes nausea and emesis via activation of prostanoid receptors. Prostaglandins and thromboxane A_2_ are lipid-mediators that play a major role in physiological and pathological processes. Five major types of prostanoid receptors have been identified: DP, EP, FP, IP, and TP (Coleman et al., 1994). Prostanoids acting on DP, EP, and TP receptors are associated with emesis in animals and man. Sulprostone is a relatively potent EP_1__/__3_ receptor agonist, with lower affinity FP receptors (Boie et al., 1997; Abramovitz et al., 2000). In our previous investigations, sulprostone dose-dependently induced emesis in ferrets, with the response being resistant to bilateral abdominal vagotomy and conventional anti-emetic drugs (Kan et al., 2002). Given that sulprostone also causes diarrhea, we reasoned that it would be an ideal exemplar to cause gastric changes so that we could compare different analytical approaches to the assessment of normal and disturbed gastric rhythm.

## Materials and Methods

### Animals

Eight castrated male fitch ferrets (1.68 ± 0.06 kg) were purchased from Southland Ferrets (Invercargill, New Zealand) and housed in a temperature-controlled room at 24°C ± 1°C under artificial lighting, with lights on between 06:00 and 18:00. The relative humidity was maintained at 50% ± 5%. Water and food (TriPro super premium chicken meal formula dog food, American Nutrition, United States) were given *ad libitum* unless otherwise stated. All experiments were conducted under license from the Government of Hong Kong Special Administrative Region and the Animal Experimentation Ethics Committee of The Chinese University of Hong Kong.

### Implantation of Radiotelemetric Devices

Eight ferrets were fasted overnight but allowed free access to water. The surgical procedures for implanting radiotelemetric devices (C50-PXT; Data Sciences International, United States) have been described in our previous studies (Tu et al., 2017). Briefly, in the anesthetized animals, following a midline abdominal incision, a 19G needle was used to pierce the aorta, and then the catheter of a C50-PXT transmitter (Data Sciences, Inc, United States) was inserted up to a length of approximately 2 cm. A 2 × 2 mm piece of sterile gauze was placed over the catheter’s entry point, and fixed with a drop of tissue glue. The body of the transmitter was then sutured to the left side of the ferret’s abdominal wall muscle with the biopotential wires and catheter facing caudally. The gastric antrum was exposed and the biopotential wires were inserted into the muscle and secured in place by suturing the serosa. The abdominal cavity was sutured in closed layers and covered with a permeable spray dressing (Opsite^®^, Smith and Nephew, United Kingdom). After the surgery, animals were allowed to recover for 7 days before drug treatment.

### Measurement of the Effect of Sulprostone [30 μg/kg, Intraperitoneal (i.p.)] on Emesis Behaviors, GMA, Cardiovascular Homeostasis and Core Body Temperature

The animals were divided into two groups (*n* = 4 for each group) and were randomized to receive a single treatment of saline (1 mL/kg, i.p.) or sulprostone (30 μg/kg, i.p.). At 08.00, the ferrets were presented with 20 g of food and allowed to eat. At 08.30, any uneaten food was removed and the animals were fasted again. At 10.00 (*t* = 0), they were injected with either sterile saline (1 mL/kg, i.p.) or sulprostone (30 μg/kg, i.p.). Their behavior was then recorded for 4 h. Radiotelemetry of cardiovascular parameters, core body temperature and GMA were recorded throughout the experiment using Dataquest ART 4.1 (Data Sciences International, United States). At the end of the experiments, the ferrets were euthanized using an overdose of pentobarbitone (80 mg/kg, i.p.).

### Measurement of Emesis and Defection/Tenesmus

After sulprostone injection, behavior was recorded for 4 h via a video system (Panasonic WV-PC-240, China). Emesis was characterized as rhythmic abdominal contractions that were either associated with the oral expulsion of solid or liquid material from the gastrointestinal tract (i.e., vomiting), or not associated with passage of material (i.e., retching). An episode of retching and/or vomiting was considered separate when the animal changed its location in the observation cage, or when the interval between retches and/or vomits exceeded 5 s (Rudd et al., 1994). An episode of defecation was characterized as a series of lower abdominal contractions and expulsion of solid or liquid fecal matter from the anus. Episodes of tenesmus were characterized as non-productive lower abdominal contractions; only tenesmus involving spasm of the anal sphincter was recorded to differentiate tenesmus from non-productive attempts of the animals to micturate. Furthermore, the animals had raised tails during all episodes of defecation and tenesmus, and episodes were considered separate when the animal changed its location in the observation cage (Kan et al., 2017).

### Data Analysis of Telemetric Recordings

All telemetric data were processed using Spike2 (Version 7; Cambridge Electronic Design). The method of telemetric GMA data analysis has been described in previous studies; dominant power was defined as the highest power in the 0–15 cpm range, and DF was defined as the frequency bin with the highest power in the 0–15 cpm range; the definition of brady-, normo-, and tachy-gastria ranges were as follow: bradygastria: 0 to (DF - 1) cpm, normogastria: DF ± 1 cpm, tachygastria: (DF + 1) to 15 cpm (Percie du Sert et al., 2009b). Systolic BP was calculated from the peak of the BP recording trace, while diastolic BP was calculated from the trough of the BP recording trace. To measure HR (in bpm), the peak-to-peak interval was first calculated using the following formula: HR = 60/P-P interval. The standard deviation of the R-R intervals (SDNN) was measured based on the P-P wave interval of the BP in 5-min segments. It was then used as an index of HR variability (HRV) (Lu et al., 2017a). Frequency-domain analysis was performed using fast Fourier transform (FFT). The total power of all R-R intervals in 5-min segments was determined, along with its low-frequency component (LF: 0.04–0.15 Hz), high-frequency component (HF: 0.15–0.7 Hz) and LF/HF ratio (Lu et al., 2017c). Core body temperature data were calculated by averaging the data. All data were averaged in 1-h segments for statistical analysis.

### Detrended Fluctuation Analysis

To carry out DFA, the GMA data were subjected to the following formulas (Peng et al., 1994; Kantelhardt et al., 2001): By dividing the cumulative sum ${\left\{y_{t}\right\}}_{t=1}^{N}$ of the given series ${\left\{x_{t}\right\}}_{t=1}^{N}$ to *N*_*s*_ = *N*/*s* non-overlapping segments with length *s*, the variance for ν-th segment can be calculated to measure the fluctuations around the *m*-th order polynomial local trend *p*_*t*_(*v*) as $f^{2}\,\left(\nu ,s\right)=\frac{1}{s}\,\sum_{t=1+\left(\nu -1\right)\,s}^{\nu \,s}{\left(y_{t}-p_{t}\,\left(v\right)\right)}^{2}.$ The fluctuation function *F*(*s*) at the scale *s* can be obtained as $F^{2}\,\left(s\right)=\frac{1}{N_{s}}\,\sum_{\nu =1}^{N_{s}}f^{2}\,\left(\nu ,s\right)$. The scaling behavior of *F*(*s*) can be obtained with varying scale *s*. The code for DFA analysis in the MATLAB software was provided in the Supplementary Material. Typically, DFA focuses on the DFA exponent *h*, which assumes the power-law asymptotic behavior *F*(*s*)∼*s*^*h*^, because it can be connected to the auto-correlation of a time series. In principle, the segmented statistical equivalence is a crucial condition of DFA validation; this implies that the fluctuations around the local trend were statistically distinguishable in all segments (Höll et al., 2016). It is easy to see that the DFA exponent *h* only extracts part of such information: the power of the scaling behavior of the fluctuation function *F*(*s*). As seen from its calculation, the fluctuation function *F*(*s*) contains rich information about the fluctuations around the local trends at each *s*, which plays a role similar to that of variance in probability theory. In the general sense, direct consideration of *F*(*s*) has two advantages for characterizing the difference between two series. On one hand, only for the series with the fluctuation function *F*(*s*) following the power law, it is possible to estimate the DFA exponent. On the other hand, taking two series *z*_*1*_ and *z*_2_with the same DFA exponent *h*_1_ = *h*_2_, their fluctuation functions can be expressed as *log*⁡*F*_1_(*s*) = *a*_1_ + *h*_1_*log*⁡*s* and *log*⁡*F*_2_(*s*) = *a*_2_ + *h*_2_*log*⁡*s*. Although the DFA exponent cannot distinguish these two series because of the same value, the fluctuation functions *F*_1_(*s*) and *F*_2_(*s*) may do so if *a*_1_≠*a*_2_. Therefore, in this study we prefer the fluctuation function *F*(*s*) to the estimated DFA exponent *h* for characterizing the given signals. However, we by no means imply that the DFA exponent is not important. We only want to extract useful information from the DFA fluctuation function when its scaling behavior is not a power law, which makes it impossible to estimate the DFA exponent.

### Sample Entropy Analysis

To capture information about irregularity at multiple scales, multiscale entropy (MSE) analysis, which is based on SampEn, has been proposed (Costa et al., 2002).

For a given time series ${\left\{x_{t}\right\}}_{t=1}^{N}$, MSE analysis calculates SampEn(*m*,*r*,*s*) at the concerned scales, *s*. The computation procedure and the meaning of the parameters *m*, *r*, and *N*_τ_ are as follows (Costa et al., 2002): By coarsening the given series ${\left\{x_{t}\right\}}_{t=1}^{N}$ at a given scale *s* to construct ${\left\{y_{t}\,\left(s\right)\right\}}_{t=1}^{N_{s}}$ as $y_{t}\,\left(s\right)=\frac{1}{s}\,\sum_{i=\left(t-1\right)\,s+1}^{t\,s}x_{t}$, where $N_{s}=\frac{N}{s}$, the regularity of ${\left\{x_{t}\right\}}_{t=1}^{N}$ at the scale *s* can be obtained as $\text{SampEn}\,\left(m,r,s\right)=-\ln\left(\frac{A\,\left(m,r\right)}{B\,\left(m,r\right)}\right)$. By respectively defining the embedded vectors ${\vec{y}}_{j}\,\left(m\right)=\left\{y_{j}\,\left(s\right),y_{j+1}\,\left(s\right),\ldots ,y_{j+m-1}\,\left(s\right)\right\}$ with *j* = 1, 2,…,*N*_*s*_−*m* + 1 and ${\vec{y}}_{j}\,\left(m+1\right)=\left\{y_{j}\,\left(s\right),y_{j+1}\,\left(s\right),\ldots ,y_{j+m}\,\left(s\right)\right\}$ with *j* = 1, 2,…,*N*_*s*_−*m*, *A*(*m*,*r*) and *B*(*m*,*r*) is the probability of observation of pairs of ${\left\{{\vec{y}}_{j}\,\left(m\right)\right\}}_{j=1}^{N_{s}-m+1}$ and ${\left\{{\vec{y}}_{j}\,\left(m+1\right)\right\}}_{j=1}^{N_{s}-m}$with the distance less than *r*×SD, where SD is the standard deviation of the series. In formula, $A\,\left(m,r\right)=\frac{1}{N-m}\,\sum_{i=1}^{N_{s}-m}\frac{\#\,\left\{j|d\,\left({\vec{y}}_{i}\,\left(m\right),{\vec{y}}_{j}\,\left(m\right)\right)\leq r\times \text{SD},\,j\neq i\right\}}{N-m-1}$ and $B\,\left(m,r\right)=\frac{1}{N-m}\,\sum_{i=1}^{N_{s}-m}\frac{\#\,\left\{j|d\,\left({\vec{y}}_{i}\,\left(m+1\right),{\vec{y}}_{j}\,\left(m+1\right)\right)\leq r\times \text{SD},\,j\neq i\right\}}{N-m-1}$. Where # denotes the set cardinality and *d* is the distance function defined as $d\,\left({\vec{y}}_{i}\,\left(m\right),{\vec{y}}_{j}\,\left(m\right)\right)=\max_{0\leq \text{k}\leq \text{m}-1}\left|y_{i+k}\,\left(m\right)-y_{j+k}\,\left(m\right)\right|$.

The code for SampEn analysis in the MATLAB software was provided in the Supplementary Material.

### Statistical Analysis

All data analyses were performed using GraphPad Prism version 6 (GraphPad Prism Software, La Jolla, CA, United States) and MATLAB (MathWorks, Natick, MA, United States). Differences in emesis and defecation between treatment groups were assessed using unpaired Student’s *t*-tests, as appropriate. Differences in BP, HR, HRV, core body temperature, GMA, DFA and entropy were assessed using repeated-measures two-way ANOVA and Bonferroni tests. All data are expressed as mean ± standard error of the mean. Differences were considered statistically significant at *P-*values < 0.05.

### Drug Formulation

Sulprostone (Sigma-Aldrich, St. Louis, United States) was dissolved in saline (0.9% w/v) and administered in a volume of 1 mL/kg, i.p.

## Results

### The Action of Sulprostone to Induce Emesis and Defecation/Tenesmus in Ferrets

**FIGURE 1:**
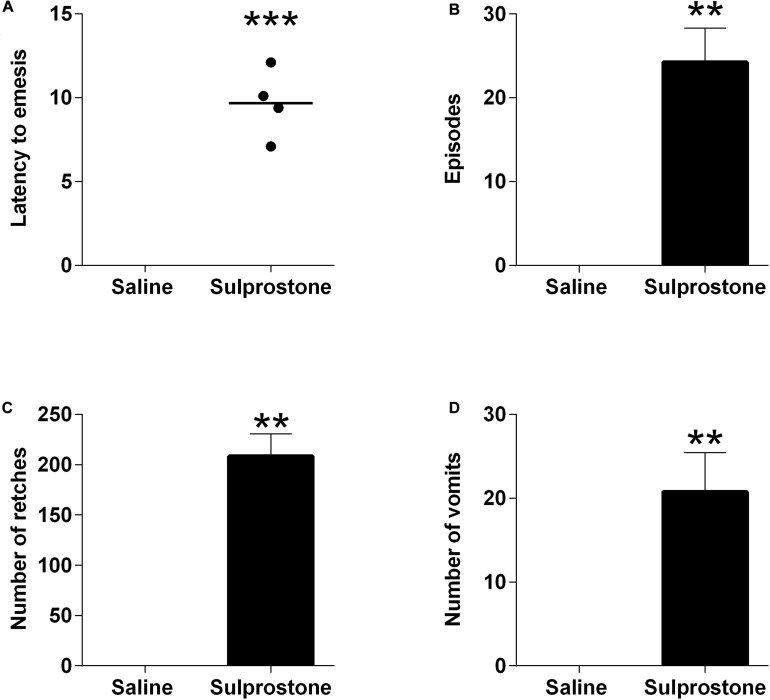
Effect of intraperitoneal administration of sulprostone (30 μg/kg) or saline (1 mL/kg) in the ferret. **(A)** Latency to the first emetic episode. **(B)** Number of episodes. **(C)** Number of retches. **(D)** Number of vomits. The episodes, retching and vomiting data are represented as mean ± SEM, using four animals. Latency data are shown as individual responses, with lines indicating the median response. Significant differences compared to the saline control group are indicated as **P < 0.01 and ***P < 0.001 (unpaired Student’s *t*-test).

The i.p. administration of saline did not induce either retching or vomiting in any of the animals tested, but it was associated with 2.3 ± 0.8 episodes of defecation. Sulprostone at 30 μg/kg, i.p. induced emesis in all ferrets. The emetic response comprised 208.5 ± 22.3 retches and 27.8 ± 4.7 vomits, following a median latency of 9.8 min (Figure 1; *P* < 0.05). Sulprostone also induced 6.8 ± 0.9 episodes of defecation/tenesmus, with a median latency of 10.7 min (*P* < 0.05, compared to saline-treated animals).

### Power Partitioning of GMA and Application of RSA

**FIGURE 2:**
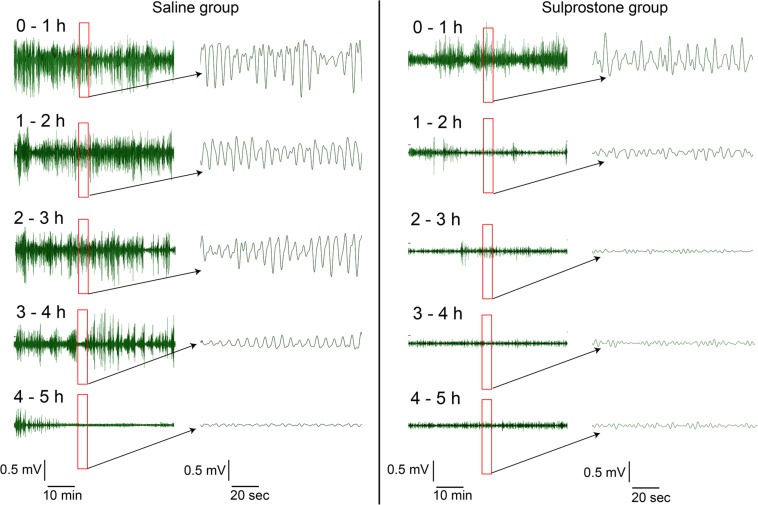
Representative traces of gastric myoelectric activity in a ferret that had received i.p. administration of sulprostone (30 μg/kg) or saline (1 mL/kg). Visual inspection of the GMA signal shows that sulprostone decreases the power of the signal, but increases the number of oscillations.

**FIGURE 3:**
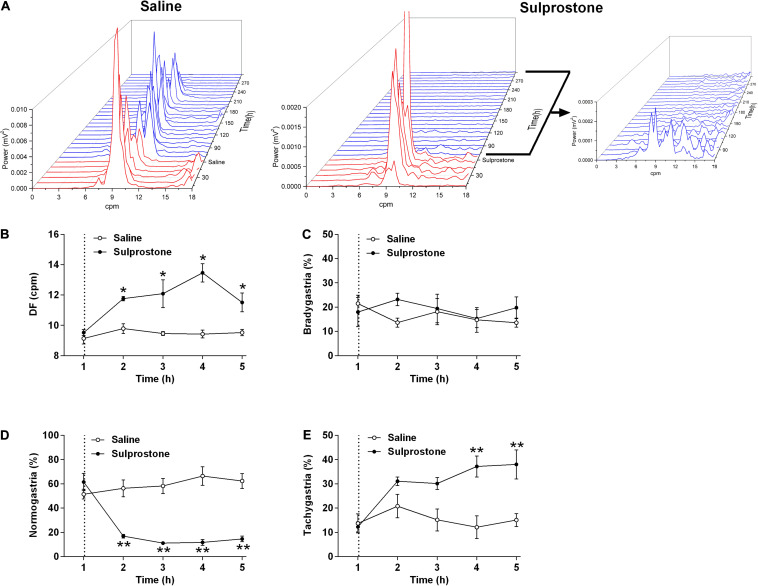
Effect of i.p. administration of sulprostone (30 μg/kg) or saline (1 mL/kg) on gastric myoelectric activity (GMA), as determined using running spectral analysis in the ferret. **(A)** RSA of GMA from the start of baseline recording 1 h prior to sulprostone or saline administration to 4 h after sulprostone or saline administration. **(B)** DF, **(C)** bradygastria (%), **(D)** normogastria (%), and **(E)** tachygastria (%) in animals that had received sulprostone or saline. Dashed line: injection of sulprostone or saline. Data represent the mean ± SEM of four animals. Significant differences compared to the saline control group are indicated as *P < 0.05 and **P < 0.01 (repeated-measures two-way ANOVA).

Visual comparison of the GMA traces with traces obtained from saline-treated animals showed that sulprostone tended to reduce the power of the signal and disrupted the normal rhythm (Figure 2). RSA revealed that sulprostone diminished the dominant power, and that the DF increased toward the tachygastric range, whereas saline had little effect (Figure 3A). Thus, baseline GMA recordings (0–1 h) revealed a DF of 9.3 ± 0.2 cpm (expected normal DF: ∼9.0-9.4; Percie du Sert et al., 2009a; Lu et al., 2017a), whereby 19.7% ± 3.3% of power was in the bradygastric range, 56.5% ± 4.2% was in the normogastric range, and 13.2% ± 2.1% was in the tachygastric range (pooled data, *n* = 8; Figure 3). Saline did not affect any of the GMA parameters, but sulprostone increased the DF significantly by approximately 2.6 cpm (DF with saline = 9.6 ± 0.2 cpm vs. DF with sulprostone = 12.2 ± 0.3; Figure 3A). Furthermore, sulprostone conferred a 77.6% decrease in the percentage power of normogastria and a 115.8% increase in the percentage power of tachygastria when compared with saline control; these effects persisted for approximately 4 h (Figures 3D,E). The percentage power of the bradygastric range was unaffected (Figure 3C).

### Detrended Fluctuation Analysis

**FIGURE 4:**
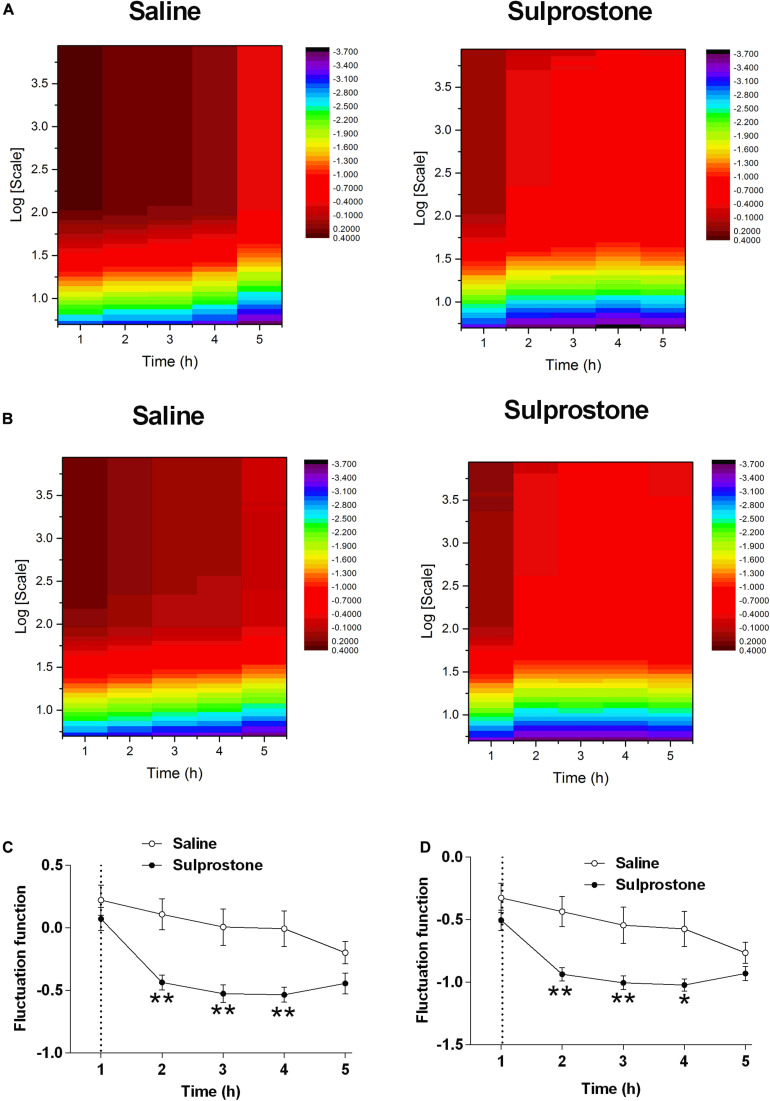
Effect of i.p. administration of sulprostone (30 μg/kg) or saline (1 mL/kg) on gastric myoelectric activity (GMA), as determined using detrended fluctuation analysis (DFA) in the ferret. **(A)** DFA fluctuation function of GMA in one sample ferret that had received i.p. administration of sulprostone (30 μg/kg) or saline (1 mL/kg). For each scale *s* and each time *h*, the fluctuation functions *F*(*s*) of the corresponding sample can be calculated. For example, the value of the heatmap corresponding to h = 1 and *log*⁡*s* = 1 indicates *F*(*s* = 10) of the sample collected at *h* = 1. **(B)** Averaged DFA fluctuation function of GMA over all samples in the sulprostone and saline groups. For example, the value of the heatmap corresponding to *h* = 1 and *log*⁡*s* = 1 indicates the averaged *F*(*s* = 10) over all samples in each group collected at *h* = 1. **(C)** Averaged DFA fluctuation function of GMA over all samples in each group at one scale of 28, i.e., *F*(*s* = 28). **(D)** DFA fluctuation function of GMA averaged over all scales, i.e., $\bar{F}$. Dashed line: injection of sulprostone or saline. Data represent the mean ± SEM of four animals. Significant differences compared to the saline control group are indicated as *P < 0.05 and **P < 0.01 (repeated-measures two-way ANOVA).

As pointed out by Hu et al. (2001), the observed periodic trend in GMA may invalidate the power-law scaling behavior. In this case, it is meaningless to estimate the DFA exponent. Therefore, we can only directly focus on the fluctuation function *F*(*s*). We set it at a common order 2, and find with this order, the effect of sulprostone can be well identified. Therefore, it should be sufficient to use the second detrending order. The DFA fluctuation function of GMA at multiple scales was significantly lower in the sulprostone-treated animals than in the saline-treated animals (*P* < 0.05; Figures 4A,B). For example, the DFA fluctuations of GMA at a scale of 28, i.e., *F*(*s* = 28), decreased in both the saline and sulprostone-treated animals (Figure 4C). However, the effect of sulprostone to decrease the DFA fluctuations was much stronger than that of saline, while the saline effect was more persistent and lasted for a longer period. Consequently, a significant difference in DFA fluctuation between the saline and sulprostone group only occurred during the 2- to 4-h post-administration period (*P* < 0.05) and was not present after 4 h. To characterize each signal by one single indictor, we further calculate the averaged fluctuation function $\bar{F}$ as the mean of *F*(*s*) over all scales *s*. It can be seen in Figure 4D the results of $\bar{F}$ similar to that for *F*(*s* = 28) shown in Figure 4C.

### SampEn

**FIGURE 5:**
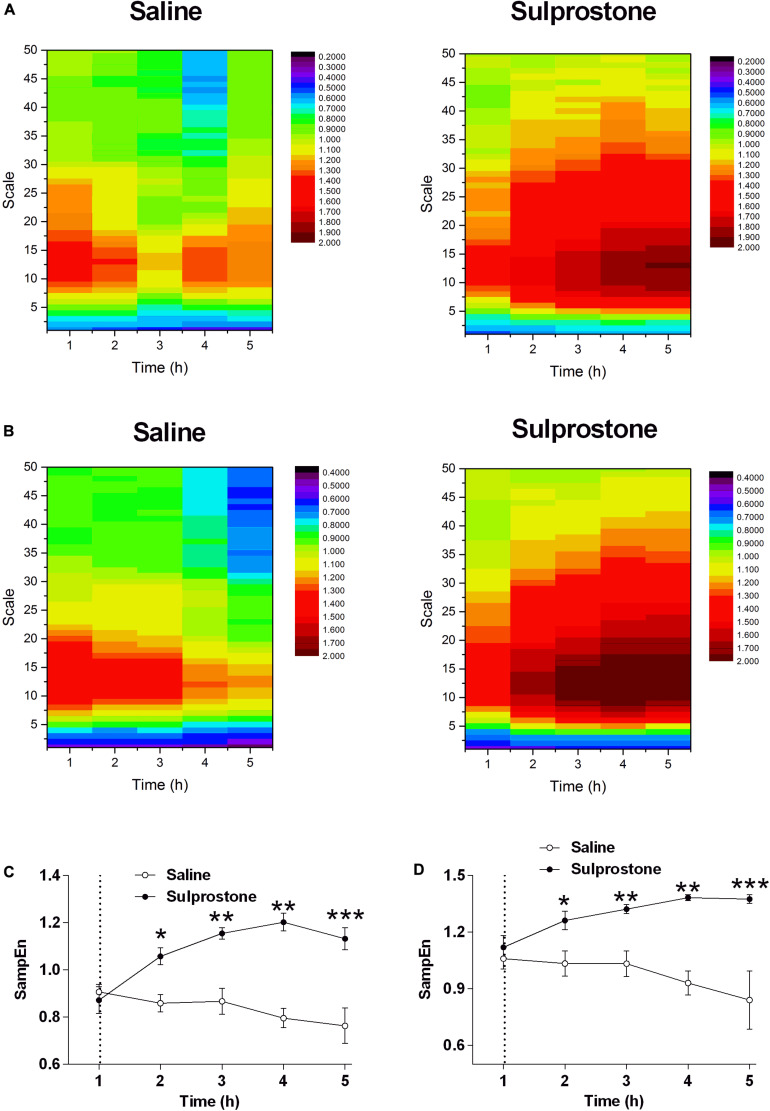
Effect of i.p. administration of sulprostone (30 μg/kg) or saline (1 mL/kg) on entropy of GMA in the ferret. **(A)** Entropy analysis of gastric myoelectric activity (GMA) in a ferret that had received an i.p. administration of sulprostone (30 μg/kg) or saline (1 mL/kg). **(B)** Averaged entropy of GMA in the sulprostone and saline groups. **(C)** Entropy analysis of GMA at a scale of 5.0. **(D)** Entropy analysis of GMA averaged over all scales. Data represent the mean ± SEM of four animals. Dashed line: injection of sulprostone or saline. Significant differences compared to the saline control group are indicated as *P < 0.05, **P < 0.01, and ***P < 0.001 (repeated-measures two-way ANOVA).

SampEn was used to evaluate the changes in signal regularity induced by sulprostone. The heatmaps shown in Figures 5A,B illustrate variations in the SampEn of GMA at multiple scales over time using one ferret and the average data from all ferrets, respectively. Generally, singular or averaged data exhibited a similar pattern: at each scale, saline decreased the SampEn over time (Figures 5C,D). In particular, at a scale of 5.0, sulprostone conferred a SampEn ∼60% than that of saline 1–4 h after administration (Figure 5C). Thus, although sulprostone increased SampEn, the increase over time appeared more gradual, and it may even have stopped or decreased 4 h after sulprostone administration.

### The Effect Sulprostone Cardiovascular Function and Core Body Temperature

**FIGURE 6:**
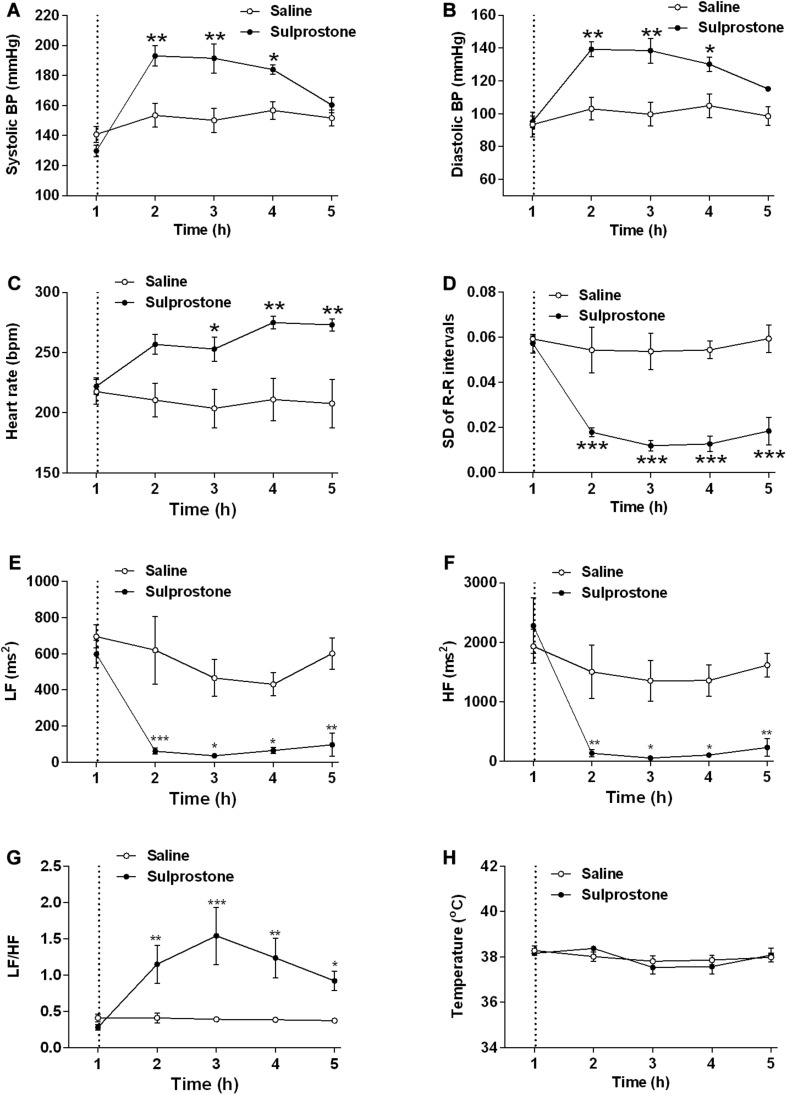
Effect of an i.p. administration of sulprostone (30 μg/kg) or saline (1 mL/kg) on cardiovascular functions and core body temperature in the ferret. **(A)** Systolic blood pressure (BP), **(B)** diastolic BP, **(C)** heart rate, **(D)** heart rate variability (HRV; SD of R-R intervals), **(E)** high frequency (HF) HRV, **(F)** low frequency (LF) HRV, **(G)** LF/HF ratio, and **(H)** core body temperature. Dashed line: injection of sulprostone or saline. Data represent the mean ± SEM of four animals. Significant differences compared to the saline control group are indicated as *P < 0.05, **P < 0.01, and ***P < 0.001 (repeated-measures two-way ANOVA).

Examination of pooled data revealed a baseline systolic BP of 135.2 ± 3.7 mm Hg and a baseline diastolic BP of 94.3 ± 3.9 mm Hg (*n* = 8; Figures 6A,B). The basal HR was 219.8 ± 5.8 bpm, and the HRV (SD of R-R intervals) was 0.058 ± 0.002 arbitrary units (Figures 6C,D). The frequency domains of the HRV were as follows: LF, 647.3 ± 49.7 ms^2^; HF, 2106.0 ± 26.15 ms^2^; and LF/HF ratio, 0.347 ± 0.038 (Figures 6E–G). The mean basal core body temperature was 38.2°C ± 0.1°C (*n* = 8; Figure 6H).

Sulprostone significantly increased systolic and diastolic BP by 19.1 and 28.9%, respectively, with significant differences observed 1–3 h after administration (Figures 6A,B); there was also a 26.9% increase in HR 2–4 h after sulprostone administration (Figure 6C). Further analysis revealed that sulprostone decreased HRV by 72.4% within 1 h of administration and the effects lasted up to 4 h (Figure 6D); it also caused a marked decrease in both the LF and HF frequency domains (Figures 6E,F), leading to an overall increase in the LF/HF ratio (Figure 6G). However, sulprostone did not affect core body temperature (Figure 6H).

## Discussion

The present study was the first to use different analytical methods to investigate the effect of sulprostone on GMA in ferrets implanted with telemetric devices. The dose of sulprostone to induce a reliable emetic response was based on our previous publications (Kan et al., 2002). Sulprostone administered peripherally predictably induced emesis and increased the frequency of defecation. This is consistent with previous studies implicating EP_1__/__3_ and FP receptors in the responses (Kan et al., 2002, 2017).

We primarily embarked on the present study to compare three analytical techniques used to assess drug action on GMA. As sulprostone induces emesis and defecation, we expected a dramatic GMA profile. In a previous study, sulprostone increased the slow wave frequency, but reduced the slow wave amplitude recorded from isolated antral muscles in mice; moreover, it increased the frequency of spontaneous inward currents from the ICCs. These effects were not prevented by SC-19220, an EP_1_ receptor antagonist, suggesting that sulprostone may affect slow wave activity via EP_3_ receptors (Kim et al., 2002). Furthermore, other studies have demonstrated that sulprostone increased the frequency of peristaltic contractions in isolated mouse antral muscles via the EP_3_ receptor (Forrest et al., 2009). In *ex vivo* perfused canine stomachs, prostaglandin E_2_ infusion increases slow wave frequency whilst decreasing the force of antral contractions (Kowalewski and Kolodej, 1975). Comparatively, data from patients with tachygastria and impaired gastric emptying have indicated that these symptoms are associated with higher levels of prostaglandin E_2_ (Sanders, 1984).

Visual inspection of the GMA signal showed that sulprostone decreased the power of the signal, but increased the number of oscillations. The slow wave signal shape became more regular, with less variation. Although the power decreased, the clustering of frequencies in the tachygastric range showed an overall increase. Indeed, conventional GMA signal processing using FFT and power spectral analysis revealed that sulprostone caused an increase in slow wave DF and reduced the power of the signals. Upon partitioning, this manifested as an increase in the percentage power of the tachygastric range and a consequent reduction in the percentage power of the normogastric range.

The detrending order is a parameter of DFA used to control the order of the fitted local polynomial trend in each segment. Mathematically, to completely remove the polynomial trend with the order *m*, the detrending order has to be at least *m+1*. Therefore, in traditional analysis, the detrending order has to be sufficiently large to ensure that the impact of local trends on the DFA scaling behavior can be completely removed. However, here we do not focus on the DFA scaling behavior and aim at fully using all information in series. It is unnecessary to completely remove the local trend. Actually, the series can be decomposed into two components at each scale, namely local trends and the fluctuations around local trends. In this sense, the DFA detrending order is not important in the traditional sense, i.e., we do not need to set it at a sufficiently large value to completely eliminate all impact of trends. Instead, we only need to see if the fluctuation around the local trends can successfully characterize the effect of sulprostone. The DFA analysis of GMA revealed that both saline and sulprostone decreased the variability of fluctuations in the GMA signals at multiple scales, indicating the loss of the signal energy over time. However, the effect of sulprostone was much greater than that of saline, and this difference was easily detected after 2–4 h. In this sense, sulprostone decreased the variability of the fluctuations, compared to the effect of saline.

In our previous studies, the analysis of GMA focused on the DF and its associated GMA power distribution. We found that the fluctuation of GMA was suppressed after cisplatin treatment in the delayed phase of emesis, while the DF of GMA was unaffected. In the present study, we applied two analytical strategies: (1) DFA and (2) MSE. DFA is used to analyze auto-correlation in either stationary or non-stationary time series, and it has been applied to various types of physiological data. In ECG analysis using DFA, pharmacological intervention that changes the sympathetic and vagal cardiac outputs (atenolol or atropine treatment) also affected the DFA scaling properties of HRV (Silva et al., 2017). It follows that DFA analysis of ECG may uncover novel implications during pathophysiological conditions. Yeh et al. (2013) pointed out that the dynamic properties of HRV revealed by DFA constitute an important feature of autonomic nervous system activity during sleep. In EEG, brain state variation, normal development, or pathological states can be detected using DFA, as can phase modulation of cortical oscillations. Furthermore, Márton et al. (2014) recently published a methodology to analyze EEG signaling using DFA. A study by Adda and Benoudnine (2016) showed that DFA of EEG achieved high detection accuracy to distinguish epileptic seizures from normal healthy EEG. To date, only limited information is available regarding DFA of GMA, probably because the time series of EGG recordings in humans have a short-duration. With the advent of radiotelemetric technology, long-term GMA from the antrum has been recorded in different species (Percie du Sert et al., 2010; Rudd et al., 2018; Wang et al., 2018). Theoretically, the observed periodic trend in GMA may invalidate the power-law scaling behavior (Hu et al., 2001). In this case, it is meaningless to estimate the DFA exponent. Therefore, we can only directly focus on the fluctuation function *F*(*s*). In the present study, the reduced DFA fluctuation of GMA was observed in sulprostone-treated animals, which may indicate reduced fluctuation of the signals. Inspection of the raw traces indicated that sulprostone caused a reduction in the GMA amplitude, perhaps because it reduced the fluctuation of GMA.

In a further analysis, we applied SampEn analysis to the ferret GMA and found that sulprostone increased the SampEn of the ferret GMA. It was difficult to distinguish the GMA irregularity by inspecting the raw traces alone. In addition, the collected GMA contains some noises. Given the advantage of SampEn for the noisy data (Richman and Moorman, 2000), it should be more appropriate to SampEn analysis rather than other entropy analysis, like ApEn. Using SampEn analysis, we showed that the SampEn of GMA was significantly higher in the sulprostone-treated than the saline-treated ferrets, and that this increase in SampEn lasted up to 4 h after sulprostone administration. In previous studies, ApEn analysis of GMA from patients with acute schizophrenia or major depression has detected increased ApEn levels and tachygastria in these patients compared with controls (Peupelmann et al., 2009; Quick et al., 2010). Taken together with our own findings, this may indicate that an elevated SampEn value points to increased complexity and dysregulation of ferret GMA after sulprostone intervention. In the present study, sulprostone reduced both the LF and HF components of HRV, with an overall increase in the LF/HF ratio, indicating a shift toward sympathetic dominance. In patients with major depression or acute schizophrenia, the increased ApEn of GMA was accompanied by an increase in sympathetic modulation, as determined by autonomic nervous symptom scores (Peupelmann et al., 2009; Quick et al., 2010). These results imply that the sulprostone-induced increase in SampEn and tachygastria is associated with an increase in sympathetic dominance, although this needs to be confirmed in further pharmacological studies. Our data inclusive of entropy and autonomic changes provide a fingerprint of prostanoid-induced changes in GI function that may underlie emesis and diarrhea. However, further studies are required to fully understand the relationships involved and if the changes are specific to EP/FP receptor activation or not.

The present study had several limitations. First, the relatively small sample size made it difficult to correlate the measured parameters, including DF, DFA and SampEn, with the severity of the emetic response. Second, it is not yet clear whether the sulprostone-induced changes in DF, DFA, and SampEn could be pharmacologically reversed by appropriate antagonists. Third, measurement of GMA using few electrodes has been demonstrated to be unreliable at capturing all types of gastric dysrhythmias (O’Grady et al., 2012), and future studies may consider multiple electrodes to investigate the spatial patterns of GMA. It would also be interesting to apply the analytical techniques identified in the present study to datasets generated using multielectrode arrays.

## Conclusion

In conclusion, the present study revealed that sulprostone induced emesis, disrupted GMA and modulated cardiovascular function in ferrets. A further comprehensive analysis in GMA using RSA, DFA, and SampEn measures showed that sulprostone induced tachygastria, decreased GMA fluctuation and increased the SampEn level of GMA, suggesting loss of variability around local trends, increased multifractality and GMA irregularity. Further studies involving other emetic stimuli such as cisplatin are needed to clarify the biological significance of DFA and SampEn in GMA analysis.

## Data Availability Statement

The original contributions presented in the study are included in the article/Supplementary Material, further inquiries can be directed to the corresponding author/s.

## Ethics Statement

The animal study was reviewed and approved by All experiments were conducted under license from the Government of Hong Kong Special Administrative Region and the Animal Experimentation Ethics Committee of The Chinese University of Hong Kong.

## Author Contributions

JR conceived the study and was in charge of overall direction and planning. ZL performed the animal surgery, experiments, and data collection. YZ performed the DFA and entropy analysis of GMA and interpreted the results. MN recorded the animal behaviors. ZL, LT, SC, YL, IH, JK, CH, and JR finalized the manuscript. All authors reviewed the final version of the manuscript and approved submission.

## Conflict of Interest

The authors declare that the research was conducted in the absence of any commercial or financial relationships that could be construed as a potential conflict of interest.
