# Supplementary material for: Sulprostone-Induced Gastric Dysrhythmia in the Ferret: Conventional and Advanced Analytical Approaches
Source: Front Physiol. 2021 Jan 8;11:583082. doi: 10.3389/fphys.2020.583082 (PMC7820816; doi:10.3389/fphys.2020.583082)
Supplement: Supplementary file 2 [file Image_1.pdf]

Code for DFA analysis

```
function mypara=MFDFA(X,m,q)


N=size(X,1);


X_ave=sum(X)/N;
Y=zeros(1,N);
for i=1:N
    Y(i)=sum(X(1:i)-X_ave);
end
num_s=60;
F_q=zeros(num_s,1);
s1=zeros(num_s,1);
for i=1:num_s
    s=floor(10^(log10(35000/20)/num_s*i+log10(5)));
    s1(i)=s;
    N_s=fix(N/s);
    F=zeros(1,2*N_s);
    for v=1:N_s
        p=polyfit([1:s],Y((v-1)*s+1:v*s),m);
        y=polyval(p,[1:s]);
        F(v)=sum((Y((v-1)*s+1:v*s)-y).^2)/s;
    end
    for v=N_s+1:2*N_s
        p=polyfit([1:s],Y(N-(v-N_s)*s+1:N-(v-N_s-1)*s),m);
        y=polyval(p,[1:s]);
        F(v)=sum((Y(N-(v-N_s)*s+1:N-(v-N_s-1)*s)-y).^2)/s;
    end
   F_q(i)=(sum(F.^(q/2))/(2*N_s))^(1/q);
end
mypara=[log10(s1) log10(F_q)];
```

Code for SampEn analysis

```
function mypara=phi(m,r,x)

%x should be column vector


N=length(x);

%reconstuct the phase space with dimension m

Nm=N-m+1;

u=zeros(Nm,m);

for i=1:m

    u(:,i)=x(i:(Nm+(i-1)));

end

C=zeros(Nm,1);

for i=1:Nm

    U_m=max( abs(u-ones(Nm,1)*u(i,:)), [], 2);

    ind=find(U_m<r);

    C(i)=size(ind,1)/Nm;

end

mypara=sum(log(C))/Nm;


function mypara=ApEn(m,r,x)


mypara=phi(m,r,x)-phi(m+1,r,x);
```
